# Multi‐phase failure modes and effects analysis for low dose bilateral whole lung irradiation of COVID‐19 positive patients requiring respiratory ventilation

**DOI:** 10.1002/acm2.14261

**Published:** 2024-01-09

**Authors:** Amanda E. Swanson, Dominic J. DiCostanzo, Nilendu Gupta, Kathleen Hintenlang, Arnab Chakravarti, Ashley J. Cetnar

**Affiliations:** ^1^ Department of Radiation Medicine Oregon Health & Science University Portland Oregon USA; ^2^ Department of Radiation Oncology The Ohio State University Columbus Ohio USA

**Keywords:** COVID‐19, FMEA, radiotherapy, risk assessment

## Abstract

**Purpose:**

To identify high‐priority risks in a clinical trial investigating the use of radiation to alleviate COVID‐19 pneumonia using a multi‐phase failure modes and effects analysis (FMEA).

**Methods:**

A comprehensive FMEA survey of 133 possible causes of failure was developed for the clinical trial workflow (Phase I). The occurrence, severity, and detection risk of each possible cause of failure was scored by three medical physicists. High‐risk potential failure modes were identified using the risk priority number (RPN) and severity scores, which were re‐scored by 13 participants in radiation oncology (Phase II). Phase II survey scores were evaluated to identify steps requiring possible intervention and examine risk perception patterns. The Phase II participants provided consensus scores as a group.

**Results:**

Thirty high‐priority failure modes were selected for the Phase II survey. Strong internal consistency was shown in both surveys using Cronbach's alpha (*α*_c_ ≥ 0.85). The 10 failures with the largest median RPN values concerned SARS‐CoV‐2 transmission (*N* = 6), wrong treatment (*N* = 3), and patient injury (*N* = 1). The median RPN was larger for COVID‐related failures than other failure types, primarily due to the perceived difficulty of failure detection. Group re‐scoring retained 8/10 of the highest‐priority risk steps that were identified in the Phase II process, and discussion revealed interpretation differences of process steps and risk evaluation. Participants who were directly involved with the trial working group had stronger agreement on severity scores than those who were not.

**Conclusions:**

The high ranking of failures concerning SARS‐CoV‐2 transmission suggest that these steps may require additional quality management intervention when treating critically ill COVID‐19+ patients. The results also suggest that a multi‐phase FMEA survey led by a facilitator may be a useful tool for assessing risks in radiation oncology procedures, supporting future efforts to adapt FMEA to clinical procedures.

## 1 INTRODUCTION

Low dose radiation has historically been used to treat severe pneumonia symptoms.^1^ Consequently, low dose radiation treatment has been proposed as an effective method to quickly alleviate symptoms of acute respiratory distress syndrome caused by COVID‐19 and improve outcomes for these critically ill patients. To evaluate the efficacy of low dose whole lung radiotherapy on patients requiring mechanical ventilation, a phase II clinical trial which uses a single fraction 80 cGy photon therapy dose delivered to the lungs was implemented, titled “VENTED” (NCT04427566). The trial is being conducted by a small team of therapists, physicians, and physicists, and has been reviewed by the institutional review board at The Ohio State University (#2020H0197).

Due to the accelerated timing of planning and delivery required by the critically ill status of the patients, infrequent occurrence of patient enrollment, potential variations in treatment delivery (such as patient positioning), high transmission likelihood of SARS‐CoV‐2, and atypical workflow for this study, it is useful to preemptively evaluate risks present in the trial procedure. Analyzing these risks would benefit not only those working on the current trial but could potentially assist other institutions that choose to implement similar protocols.

The purpose of this study is to apply a streamlined Failure Modes and Effects Analysis (FMEA) to a novel radiation oncology workflow to identify high‐priority risks and evaluate risk perception patterns concerning failures related to SARS‐COV‐2 transmission and treatment errors. An FMEA requires participants to evaluate three aspects of each potential failure mode: the likelihood of occurrence (O), the severity of the failure if it is not detected (S), and the likelihood that the failure will not be detected in time to avoid adverse effects, or “detection risk” (D). This aspect (D) is often labeled “detection”, but was changed to “detection risk” to avoid confusion when assigning scores: a high “detection” score may imply that something is easy to detect, so the label was changed to clarify that a higher score corresponds to a greater risk of being undetected. Each risk category (O, S, and D) is assigned a score on a scale from 1 to 10, where increasing scores correspond to increasing risks. The product of the resulting scores is then used to determine the “risk priority number” (RPN), where larger RPNs correspond to higher‐priority risks. This format is increasingly used for prospective risk analysis for quality management within Radiation Oncology^2^, ^3^ and to mitigate risks for various treatment workflows.^4^, ^5^, ^6^, ^7^, ^8^ It has also been applied to specifically characterize risks associated with SARS‐CoV‐2 transmission in literature.^9^ Other works have also customized the FMEA workflow recommended by the American Association of Physicists in Medicine Task Group 100′s report on the application of risk analysis methods to radiation therapy quality management (AAPM TG 100) to reduce the time commitment and training requirements for participants.^10^

In this study, a comprehensive FMEA framework was assembled by a project facilitator, and the risk assessment was remotely completed in two phases. Phase I was a comprehensive assessment of the trial workflow completed by a small team of physicists directly involved in the development of the trial to identify high‐priority risk failure modes and exclude low‐priority steps from the analysis. The results of the Phase I survey were applied towards creating a more focused survey (Phase II), which was evaluated by radiation oncology professionals in the VENTED working group, including physicians and therapists, as well as a larger cohort of medical physicists at our institution. Participants also evaluated the Phase II survey as a group to determine consensus risk scores, which were compared to the results obtained by individual evaluations.

## 2 METHODS

**FIGURE 1:**
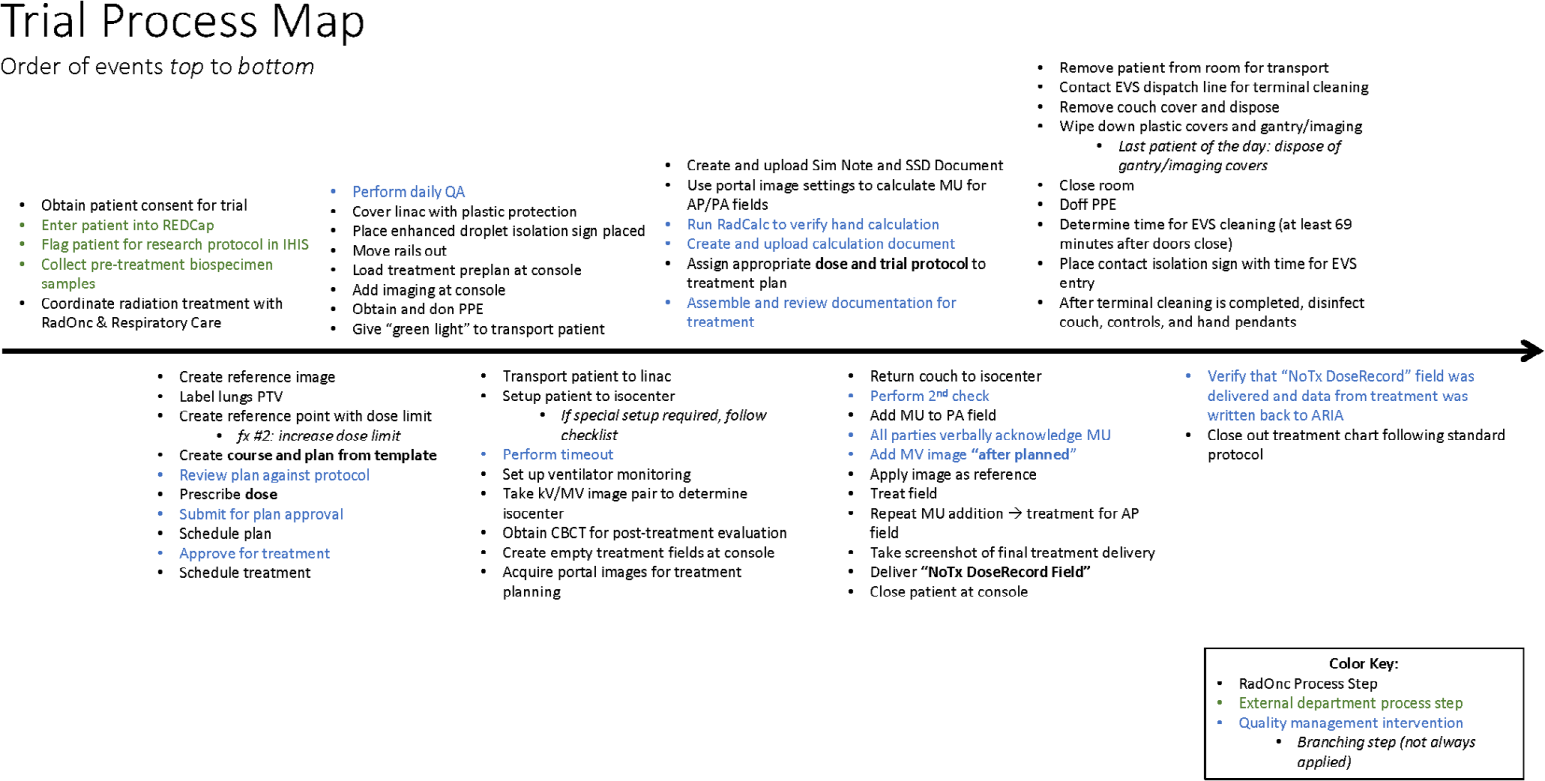
Process map adapted from the written clinical workflow for the trial. Process steps that are not in radiation oncology are marked green, existing quality management interventions are marked blue, and conditional steps that are not always present are indented and italicized.

Risks in the VENTED trial procedure were identified by creating a process map, shown in Figure 1. Treatment was delivered using a “sim‐and‐treat” process on a Varian TrueBeam, with additional steps and precautions to prevent staff exposure to SARS‐CoV‐2 and ensure the safety of the ventilated patient during treatment. The original written procedure was drafted by an interdisciplinary team in the trial working group, including physicians, physicists, and radiation therapists. A process map was adapted from the written procedure by two medical physicists, one of whom was actively involved in the VENTED working group while the other acted as the FMEA facilitator. The process map focuses on the Radiation Oncology department's role in the clinical trial, and the steps are organized into seven sections (from ‘patient selection and preparation’ to ‘post‐treatment’). To accurately reflect the entire protocol, explicit quality management interventions and coordination with departments external to radiation oncology were included in the process map. The facilitator assigned potential failure modes with corresponding potential causes of failure to each process step. Each cause of failure was assigned a corresponding ‘end effect’ to assist with categorization, such as an incorrect treatment delivery or breach of protected health information. The resulting FMEA framework was organized using a formatting tool provided by AAPM TG 100 and Beth Israel Lahey Health Radiation Oncology.^11^

The Phase I FMEA was constructed to be easily understood and completed by a radiation oncologist, physicist, or therapist familiar with the trial procedure. A limited number of end effects were included in the survey to quickly communicate the intent of the facilitator while permitting each respondent to apply their best judgment to each failure mode, described in Table 1. Additionally, the facilitator provided a short guide to all participants that included instructions for use and a simplified scoring guide for each risk category informed by the recommended risk stratification in AAPM TG 100.^12^ This scoring guide did not include a severity scale specifically for end effects concerning viral transmission. RPN scores were initially determined using the average O, S, and D scores from the participants, but the median was employed during Phase II to better represent data on an ordinal scale with unknown biases. For clarity, the median value is reported in this study for both phases.

**TABLE 1:** End effects applied in Phase I VENTED trial FMEA with brief descriptions provided to survey respondents.

End effect	Description
Wrong treatment	Wrong dose, dose distribution, location, or volume *Encompasses all severity levels, to be determined by FMEA working group*
Wrong patient	Wrong patient treated for radiation
COVID‐19 exposure	COVID‐19 droplet exposure
COVID‐19 contamination	COVID‐19 contamination on surfaces
Patient injury	Non‐radiation harm to patient
Collision	Mechanical collision on linac which may or may not harm the patient
Treatment delay	Delay in radiation treatment
Unintended radiation exposure	Unnecessary radiation exposure to patient or personnel
PHI breach	Misuse or disclosure of protected health information
Inaccurate record	Record error that may impact plan review (missing information or misrepresentation of treatment)
Inconsistent record	Record error that is inconsistent with standard practice but does not directly impact patient care

Abbreviation: FMEA, failure modes and effects analysis.

Following the initial survey results, the Phase II survey was created to focus on failure modes that may require quality management intervention and increase the number of scoring participants. The following criteria determined which failure modes would be included in the Phase II survey:

1. Failure mode is ranked among the *X* highest average RPN on the initial survey (where *X* is a cutoff value determined by the results of the initial survey);
2. Failure mode has an average severity ranking of 7.5 or higher;
3. If fewer than three process steps in any section meet criteria 1 or 2, the step(s) with the next highest RPN in the section is added such that at least three process steps are included for each section.

These criteria were selected to appropriately narrow the survey scope while maintaining adequate representation of the trial procedure. Feedback from the Phase I results was also applied to clarify survey contents and update training materials in Phase II, including the addition of a 15‐min virtual training session.

The final RPN ranking in Phase II was determined using the median scores from all participants, and interrater agreement was evaluated using Brown and Hauenstein's alpha coefficient (*a_wg_*), where *a_wg_* ≥ 0.80 indicates strong agreement and 0.60 ≤ *a_wg_* < 0.80 indicates moderate agreement.^12^ Cronbach's alpha (*α*_c_) is used to determine the internal consistency of surveys or tests that employ a response scale from participants,^13^ so it is used here to ensure that the Phase I/II surveys were reliably scored using the provided risk scales. Alpha values between 0.7 and 0.9 suggest appropriate internal consistency.^14^

Two months following the conclusion of the Phase II survey, participants were invited to evaluate the median O, S, and D scores for each process step and recommend changes to create a group risk assessment score. Not all participants from Phase II were able to join the group re‐scoring session, but no new participants were added during the group discussion. The group RPNs were used to re‐rank the process steps, and the top 10 steps were discussed to address possible quality management interventions. The group risk assessment is discussed to evaluate interpretation consistency and assess changes in risk assessment over time.

Following data collection, risk perception patterns among participants with different occupations and involvement with the VENTED trial were examined. Special attention was paid to differential treatment of steps involving SARS‐CoV‐2 transmission versus those involving typical radiation therapy risks.

## 3 RESULTS

The Phase I survey identified 76 potential failure modes and 133 potential causes of failure. Responses from three medical physicists in the working group were used to determine the highest‐priority risks in the FMEA. The estimated time to complete the survey was 80 min and Phase I did not require any additional time commitment from participants. The Phase II survey contained 30 possible causes of failure and took an estimated 30 min to complete. The training sessions in Phase II took approximately 15 min for each participant. The full surveys and scores for Phase I and Phase II are included as Tables S1, S2.

### 3.1 Phase I

The Phase I survey was completed by three physicists, all of whom were part of the clinical trial working group. RPN scores for Phase I were determined using the average O, S, and D scores from participants, which informed the creation of the Phase II survey, but the median values are also reported for completeness.

The internal consistency of the survey was verified using Cronbach's alpha (*α*_c_ ≥ 0.85 for O, S, and D). The highest RPN_avg_ was 204, and the average RPN was 42 (maximum RPN*_med_* = 192, median RPN*_med_* = 30). Severity had the highest average score among participants (S*_avg_* = 3.2, S*_med_* = 4). The full results of the Phase I survey are provided as a supplement (S1), which contains the average O, S, D, and RPN scores for each potential cause of failure. Due to the small number of participants, no analysis of interrater agreement is provided.

The failure modes with high severity scores included a combination of failures related to radiation therapy incidents (wrong treatment, wrong patient), patient handling (patient injury, collision), and SARS‐CoV‐2 transmission. In Phase I, ‘COVID‐19 contamination’ was scored higher on average than ‘COVID‐19 exposure’. Approximately 20% of the highest potential causes of failure (PCOF) were selected to represent the highest‐risk failures (30 PCOF).

The three highest RPN failure modes concerned SARS‐CoV‐2 transmission. The most common end effects associated with high‐risk failures were ‘wrong treatment’ (43%), ‘COVID‐19 exposure’ (30%), and ‘COVID‐19 contamination’ (17%). The protocol section containing the largest number of high‐risk failures was ‘post‐treatment’ (33%), and the section with the least high‐risk failures was ‘patient selection and preparation’ (0%). Among high‐priority risk failure modes, several related PCOF received very similar RPN scores (such as ‘time calculation forgotten’ and ‘calculation error’ as causes for the failure mode ‘incorrect entry time determined’). These steps were combined in the Phase II survey.

### 3.2 Phase II

Using the failure mode selection criteria and combining similar steps, the Phase II survey contained 30 potential causes of failure. Based on feedback from Phase I participants, the language of two end effects and several potential failure modes included were modified to increase clarity and specificity for Phase II, and multiple causes of failure that yielded similar RPN scores in the initial FMEA were combined to clarify the survey and avoid overrepresentation of similar process steps. The survey was completed by 13 participants: 2 therapists, 2 radiation oncologists, and 9 medical physicists. Five of the physicists were not directly involved with the working group but were invited to provide perspective as a skilled outside party.

The 15‐min training session provided to participants focused on communicating the risk rating scales and how they related to the survey. It included a concise overview of the contemporary public understanding of COVID‐19 transmission, risks, and sequalae at the time of training. No further guidance specific to viral transmission was provided; participants were asked to use their best judgment when scoring the severity of associated risks. The session concluded with one simple example scored by the facilitator, and one clinical example scored by the trainee.

The Phase II survey demonstrated good internal consistency (0.85 ≤ *α*_c_ ≤ 0.96 for O, S, and D). The highest RPN for the Phase II survey was 144 and the median RPN 62, demonstrating a narrower range of scores than the Phase I survey. The majority of RPNs had at least moderate interrater agreement (22/30 RPNs had *a_wg_* ≥ 0.60). Occurrence showed the most consistent interrater agreement (30/30 scores had at least moderate agreement), and severity had the least (20/30 scores showed at least moderate agreement).

**FIGURE 2:**
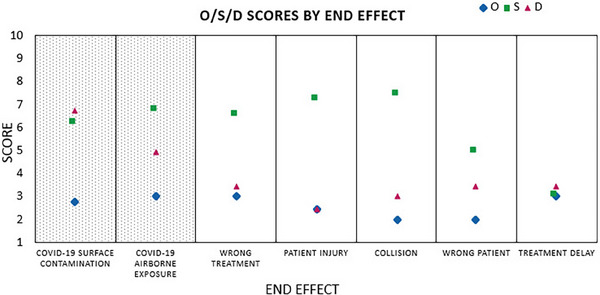
Median scores for occurrence (O), severity (S), and detection risk (D) for failure modes on the Phase II survey, grouped by end effect. Failure modes concerning COVID‐19 transmission are marked by a patterned background.

The failure modes with the 10 highest RPNs are shown in Table 2. The 6 highest‐priority PCOF concerned SARS‐CoV‐2 transmission, and the remaining 4 concerned wrong treatment delivery. Median O, S, and D scores sorted by end effect are shown in Figure 2, which highlights that the failures associated with SARS‐CoV‐2 transmission had a higher median detection risk scores (*D* = 5) than other failure types (*D* = 3), whereas the median occurrence and severity scores did not significantly differ between categories.

**TABLE 2:** Summary of the 10 highest‐priority risk failure modes identified in the Phase II survey, sorted by descending median RPN.

Process step	Failure mode	Cause of failure	End effect	O	S	D	RPN
Disinfect couch, controls, and hand pendants	Wipe down ineffective	Wipe down forgotten or performed incorrectly	COVID‐19 surface exposure	3	6	8	144
Wipe down or remove plastic covers on gantry/imaging	Plastic protection ineffective	Wipe down or removal performed incorrectly	COVID‐19 surface exposure	3	6	8	144
Contact Environmental Services dispatch for terminal cleaning	Terminal cleaning not scheduled	Environmental Services not contacted/lack of coordination	COVID‐19 surface exposure	3	6	6	108
Don/Doff PPE at treatment linac	PPE ineffective	PPE not donned/doffed appropriately, or PPE breach missed	COVID‐19 airborne exposure	3	7	5	105
Place enhanced droplet isolation sign	Sign ineffective	Sign removed too early	COVID‐19 airborne exposure	3	7	5	105
Transport patient to linac	Personnel exposed to COVID‐19 positive patient	Pathway not cleared and secured, or wrong pathway taken	COVID‐19 airborne exposure	4	6	4	96
Submit for plan approval	Error missed	Incomplete preplan review	Wrong treatment	3	6	5	90
Calculate MU for photon dose	Incorrect MU calculation	Wrong jaw settings or patient parameters used in calculation	Wrong treatment	3	7	4	84
Add MV portal image to treatment field	Positioning error missed	Image misinterpreted (error not seen)	Wrong treatment	3	7	4	84
Add calculated MU to fields	Incorrect MU entered	MU calculated or entered incorrectly	Wrong treatment	3	7	4	84

Abbreviations: D, median score for detection risk; MU, monitor units; MV, megavoltage; O, median score for occurrence; PPE, personal protective equipment; RPN, risk priority number; S, median score for severity.

The failure modes with the highest severity scores (*S* = 8) concerned patient injuries during treatment setup and transfer from the treatment couch (including ventilator compromise and gantry clearance issues). Other PCOF related to patient injury, wrong treatment, and SARS‐CoV‐2 transmission had relatively high severity scores (*S* = 7). There was no significant difference in perceived risks between surface contamination and airborne transmission of SARS‐CoV‐2.

### 3.3 Group re‐scoring risk assessment

The maximum RPN in the group re‐scoring session was 140 and the median was 57, showing good agreement with the Phase II results. The median scores for the group risk assessment compared to the individual scores in Phase II are provided as a supplementary Table S2. More RPNs were reduced (21/30 steps) than increased (9/30) during group scoring discussions, and the severity score was reduced most often (14 severity scores were decreased, whereas only 3 occurrence and detection risk scores were decreased). RPN scores for the three highest‐severity failure modes identified in Phase II (*S* = 8) were unchanged during the group re‐scoring session.

The top 10 highest‐priority risk steps determined by the group are shown in Table 3. 8 of the 10 steps were also identified as high‐priority risks in the Phase II survey (seen in Table 2), including all 6 steps related to SARS‐CoV‐2 transmission. The two process steps that were uniquely identified as high‐risk by the group concerned incorrect dose entry at the treatment console and incorrectly recording the anterior‐posterior width of the patient. Both of these steps are highlighted in Table 3.

**TABLE 3:** Summary of the 10 highest‐priority risk failure modes identified during the consensus risk assessment, sorted by descending median RPN.

Process step	Failure mode	Cause of failure	End effect	O	S	D	RPN
Doff PPE	PPE ineffective	PPE doffed too early or not doffed appropriately	COVID‐19 airborne exposure	4	7	5	140
Patient transported to linac	Personnel exposed to COVID‐19 positive patient	Pathway not clearer and secured, or wrong pathway taken	COVID‐19 airborne exposure	4	7	4	112
Don PPE at treatment linac	PPE ineffective	PPE not donned appropriately or PPE breach missed	COVID‐19 airborne exposure	4	4	6	96
After EVS: disinfect couch, controls, and hand pendants	Wipe down ineffective	Wipe down forgotten or performed incorrectly	COVID‐19 surface contamination	3	4	8	96
Submit for plan approval	Error missed	Incomplete preplan review	Wrong treatment	3	6	5	90
Assign dose to plan**	Preplan dose not 80 cGy	Dose incorrectly entered	Wrong treatment	4	7	3	84
Wipe down or remove plastic covers on gantry/imaging	Plastic protection ineffective	Wipe down ineffective	COVID‐19 surface contamination	2	6	7	84
Obtain extended CBCT**	Incorrect AP distance across lung	AP distance recorded incorrectly	Wrong treatment	3	6	4	72
Add calculated MU to fields	Incorrect MU entered	MU calculated or entered incorrectly	Wrong treatment	3	6	4	72
Contact EVS dispatch for terminal cleaning	Terminal cleaning not scheduled	EVS not contacted/lack of coordination	COVID‐19 surface contamination	2	6	6	72

*Note*: Starred process steps (**) were not identified during the Phase II process (Table 2).

Abbreviations: CBCT, cone‐beam computed tomography; D, median score for detection risk; EVS, environmental services; MU, monitor units; O, median score for occurrence; PPE, personal protective equipment; RPN, risk priority number; S, median score for severity.

Discussions during risk assessment showed that some survey items were interpreted differently by participants, which impacted how individuals evaluated the process steps in Phase II. Some steps were also re‐evaluated to reflect an updated understanding of COVID‐19 associated risks at the time of discussion. However, no systematic score changes were made for COVID‐19 related process steps.

The group re‐scoring session identified possible interventions for high‐priority risks, including assigning responsibility for process steps to a specific individual (such as designating a team leader when donning and doffing personal protective equipment, adding an additional staff member to the treatment team to coordinate patient transportation with the intensive care unit, and assigning one person to coordinate vault cleaning post‐treatment) and creating visual guides or checklists to reduce ambiguity (placing donning/doffing instructions in a visible location). These interventions were not directly applied to the clinical trial workflow due to low patient enrolment, but were used to inform future clinical workflows.

### 3.4 Demographic analysis

Demographic analysis of the Phase II survey was conducted to investigate risk perception patterns among participants. The participant demographics are shown in Table 4, and due to the small number of therapist and physician participants (*n* = 2) quantitative analysis is limited.

**TABLE 4:** Participant demographics for the Phase I/II surveys. All physicians and therapists were part of the VENTED working group.

Occupation	Phase I (*n*)	Phase II (*n*)	Members of VENTED Working Group (*n*)
Physician	0	2	2
Therapist	0	2	2
Physicist	3	9	4

The median scores and interrater agreement indices (*a*_*wg*_) for each risk category scored by participant occupation are summarized in Table 5. Occurrence and severity were scored more consistently than detection risk among all participant groups, and the physicist groups had the lowest median interrater agreement among occupation groups.

**TABLE 5:** Median scores and interrater agreement (*a*_*wg*_) for the occurrence, severity, and detection risk on the Phase II survey sorted by participant occupation.

Occupation	Occurrence *(a_wg_)*	Severity *(a_wg_)*	Detection Risk *(a_wg_)*	RPN
Therapist (*n* = 2)	2.0 (0.96)	6.0 (0.89)	2.5 (0.96)	35.5
Physician (*n* = 2)	3.0 (0.97)	6.5 (0.97)	5.5 (0.97)	90.1
Physicist (*n* = 9)	3.0 (0.81)	7.0 (0.69)	3.0 (0.72)	63.0

**FIGURE 3:**
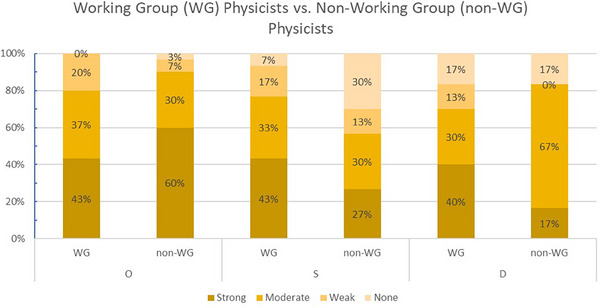
Interrater agreement of occurrence, severity, and detection scores for physicists who were directly involved in the VENTED working group and those who were not. Darker shading corresponds to increased agreement among participants in each group.

The lower interrater agreement among physicist participants was investigated further by comparing scores of those in the working group to those who were not in the working group. Figure 3 shows the proportion of scores with strong, moderate, weak, and no agreement among physicists in the working group and those who were not. This figure highlights that the physicists in the working group agreed more often than others for severity scores, but both groups showed similar agreement for occurrence and detection risks.

Phase II risk scores were also sorted by which occupation (therapist, physician, or physicist) was most responsible for the process step. In the Phase II survey, 14/30 steps were assigned to therapists, 7/30 were assigned to physicists, and the remaining steps were considered multi‐disciplinary responsibilities (such as transporting the patient to the linac from the ICU). The Phase II survey had no steps that were assigned solely to physicians.

## 4 DISCUSSION

We present the results of a streamlined FMEA as applied to a novel workflow in radiation oncology brought about by the COVID‐19 pandemic. Many failure modes identified were a direct result of working with critically ill COVID‐19 positive patients and were considered high risk, with 6 of the 10 highest potential causes of failure all concerning viral transmission. This is largely due to the high detection risk scores associated with COVID‐19 exposure. The relatively high ranking for these risks demonstrated that for the VENTED treatment protocol, the failure modes that require the most quality management intervention may be those associated with viral transmission. However, the majority of the potential causes of failure on the Phase II survey were not related to SARS‐CoV‐2 transmission (21/30), many of which would be applicable to a similar radiation oncology workflow (such as MU calculation and patient alignment).

The high priority of risks associated with nosocomial (hospital‐acquired) SARS‐CoV‐2 transmission may be influenced by the atypical precautions needed to treat patients requiring mechanical ventilation as well as the uncertainty of risks associated with COVID‐19. Scoring for these steps was necessarily informed by the rapidly developing knowledge about COVID‐19, which may have impacted risk perception patterns in the short time between phases of the study. The consistency and accuracy of the scores associated with viral transmission would have benefitted from including an expert in infectious disease pathways and associated risks, such as an epidemiologist, throughout the FMEA process. Our experience explicitly highlights the need to incorporate additional information into FMEAs, which can apply to other procedures that involve evolving procedures and technology: prior studies have demonstrated that repeating an FMEA after a time delay can significantly alter risk assessment,^7^ which can change priorities and direct mitigation strategies to process steps that require the most attention.

The remotely conducted survey tool provided a useful method for narrowing the scope of the FMEA to exclude low‐priority risk failure modes without requiring an excessive time commitment from multiple team members, which suggests that a multi‐phase remote FMEA with one or two facilitators may be feasible for evaluating procedures with relatively simple protocols. This supports other works in the literature that have employed a small team of FMEA facilitators to minimize the time commitment of the process and increase participation in a radiation oncology clinic.^4^, ^15^ Additionally, changes in high‐priority RPN ranks between the individually‐determined scores in Phase II and group re‐scoring session were minimal despite the differing scoring formats. This supports other works to suggest that individual RPN scoring can be an effective method for streamlined FMEA evaluation.^3^, ^10^ The full Phase I/II surveys are included as supplements (S1, S2) to support future quality management recommendations.

The 15‐min virtual training sessions provided to Phase II participants was well‐received, and the good agreement among Phase II participants suggests that a directed training session may be sufficient to adequate introduce participants to the FMEA scoring method. Participant training in FMEA formalism and risk assessment is encouraged by TG 100, but can be burdensome for staff members who do not routinely use these skills.^11^ This work supports the potential utility of an abbreviated training resource intended to increase participation in clinical risk assessment.

This method of employing FMEA was effective for increasing participation and removing low‐priority risk steps in this study, but limitations were identified throughout the process. While the original clinical trial workflow was written by a multidisciplinary team, the translation to a process map and initial FMEA was performed by two medical physicists. This initial process was chosen because of the facilitators’ familiarity with the FMEA process and was effective for creating a comprehensive survey, but it may have introduced bias early in the process and influenced which process steps were evaluated by the Phase II participants. The lack of physician‐led steps in the Phase II survey may have been an unintended consequence of such bias. This process also influenced the interpretation and language used for risk assessment: feedback during the group risk scoring session highlighted the importance of specific language to describe steps that can be ambiguous. Additionally, the lack of input from an expert in infectious diseases limited the accuracy of risk identification and evaluation during the FMEA process, particularly during the survey preparation and training phases. Finally, it would be beneficial to provide more opportunities for participant input early in the process to minimize the likelihood of excluding potential high‐priority risks. While the individualized survey‐based scoring method in Phases I and II encouraged participation and allowed each participant to provide full input on each process step, the consequential lack of a discussion forum introduced differences in interpretation that could have been avoided. For these reasons, the authors recommend involving a multidisciplinary team trained in formal risk assessment early in the FMEA process to minimize potential bias, improve survey clarity, and identify as many risks as possible. The formation of an interdisciplinary working group led by facilitators is a well‐established strategy in the literature and may be directly applicable to a multi‐phase survey format.^6^, ^7^, ^10^, ^15^, ^16^

## 5 CONCLUSIONS

A multi‐phase FMEA survey strategy successfully identified high‐priority risks in a novel radiation oncology workflow for treating severe COVID‐19 symptoms in patients requiring mechanical ventilation. The majority of high‐priority risks were related to nosocomial SARS‐CoV‐2 transmission, but high‐priority risks associated with typical radiation oncology treatments were also identified. The multi‐phase format increased participation with minimal additional training or time commitment from radiation oncology staff. The good agreement between the individual and group scores for the Phase II survey, as evidenced by the minimal changes to high‐priority risk rankings, support that individual scoring methods may be appropriate for identifying high‐priority risks in a radiation oncology clinical workflow.

## AUTHOR CONTRIBUTIONS

Amanda Swanson contributed to the study design, investigation, data collection, data analysis, visualization, and writing (manuscript preparation). Ashley Cetnar contributed to the conceptualization, study design, investigation, data analysis, and writing (review and editing). Dominic J DiCostanzo contributed to the investigation, data analysis, and writing (review and editing). Nilendu Gupta and Kathleen Hintenlang contributed to data analysis and writing (review and editing). Arnab Chakravarti provided resources as Principal Investigator of the clinical trial protocol.

## CONFLICT OF INTEREST STATEMENT

Arnab Chakravarti has a grant related to this work from Varian Medical Systems “(COVID‐19) Phase II protocol of low‐dose whole thorax megavoltage radiotherapy for patients with SARS‐CoV‐2 pneumonia.” Chakravarti has other additional grant funding not directly related to this work from the National Cancer Institute (R01CA188228, R01CA108633, R01CA169368, RC2CA148190, and U10CA180850‐01), Varian Medical Systems (Personalized Treatment Prediction Models based on Machine Learning Algorithms for Glioblastoma Patients and Towards the Clinical Implementation of FLASH), and the Ohio State University Comprehensive Cancer Center. Other potential conflicts of interest for Arnab Chakravarti include: Chair of the NIH Advisory Board for Radiation Oncology, NCI Advisory Board of Scientific Counselors to advise the president of the United States and other government officials in the field of CNS oncology, Chair of Radiation Oncology for OREIN Clinical Trials Network, Klotz Family Chair of Cancer Research, Chair of the Board of Directors of the International Society of Neuro‐Radiation Biology, Board of Directors of the Ohio Neuro‐Oncology Consortium, Chair of the Radiation Therapy and Oncology Group (RTOG)/NRG Brain Tumor Translational Research Subcommittee, Co‐Chair of the NRG Brain Tumor Committee, Vice Chair of the NRG Oncology Brain and Tumor Committee.

## Supporting information

Supporting Information

Supporting Information
