# Supplementary material for: Multi‐phase failure modes and effects analysis for low dose bilateral whole lung irradiation of COVID‐19 positive patients requiring respiratory ventilation
Source: J Appl Clin Med Phys. 2024 Jan 9;25(4):e14261. doi: 10.1002/acm2.14261 (PMC11005974; doi:10.1002/acm2.14261)
Supplement: Supplementary file 1 — Supporting Information [file ACM2-25-e14261-s002.pdf]

Supplemental Table 1: Average scores for the full Phase I survey.

Process Step	***Potential Failure Mode***	***Potential Cause of Failure***	***End Effect***	***O***	***S***	***D***	***RPN***
Section 1: Patient Selection and Preparation							
Obtain patient consent for trial	Contraindication for treatment missed	Incomplete history obtained	Patient injury	2.7	4.0	4.3	46.2
Enter patient in REDCap for research tracking	Wrong patient entered into REDCap	Human error	Wrong patient	2.3	9.0	1.7	35.0
Flag patient for research in IHIS	ICU team not notified that patient is part of trial	Patient not flagged for trial	Wrong treatment	2.7	3.3	3.0	26.7
Collect pre-treatment biospecimen samples	Incomplete trial data acquired	Samples not collected	Inaccurate record	2.3	2.3	2.7	14.5
Coordinate treatment time and notify teams in RadOnc and Respiratory Care	Department not notified	Text not received (no signal, device down)	Treatment delay	3.0	3.3	2.7	26.7
		Text delivered to only 1 of 2 lists	Treatment delay	2.0	3.0	2.7	16.0
	Patient transported without confirmation	Miscommunication/lack of coordination	COVID-19 exposure	2.7	3.0	2.3	18.7
	PHI delivered via text	Mix-up of personal/professional communication lines	PHI breach	4.0	3.3	1.7	22.2
Section 2: Physics Pre-Treatment							
Create reference image with PTV label	Reference image not created properly	Reference image created for wrong patient	Wrong patient	2.0	3.0	1.7	10.0
		Reference image not created	Treatment delay	2.0	2.3	1.7	7.8

Label lungs PTV	Incorrect preplan target information	PTV not created	Treatment delay	2.3	2.0	3.0	14.0
		PTV/BODY labeled incorrectly	Inconsistent record	2.3	2.0	3.0	14.0
Create reference point with dose limit	Reference point incorrect	Wrong dose limit to reference point	Wrong treatment	2.7	2.3	2.7	16.6
		Reference point assigned to location	Inaccurate record	2.7	1.7	2.0	8.9
		Reference point labeled incorrectly	Inconsistent record	2.7	1.7	2.0	8.9
Create course and plan from template	Course/plan information inaccurate	Wrong protocol selected	Wrong treatment	2.0	3.0	2.7	16.0
		Template has incorrect parameters	Wrong treatment	1.7	5.3	3.7	32.6
		Wrong energy manually selected	Wrong treatment	3.0	6.0	4.3	78.0
		Patient positioning mismatch (HFS/FFS)	Inaccurate record	1.7	4.3	2.7	19.3
Review plan against protocol	Error missed	Incorrect reference image in protocol	Wrong treatment	1.7	2.0	2.3	7.8
Prescribe dose	Preplan dose inaccurate	Dose prescribed not 80 cGy	Wrong treatment	3.0	6.3	4.0	76.0
	Preplan field mistaken as treatment field	Too many MU on preplan field	Wrong treatment	2.3	3.0	3.7	25.7
Submit for plan approval	Error missed	Warning screen misinterpreted (new warning mistaken as normal)	Wrong treatment	3.0	4.0	4.3	52.0
		Incomplete preplan review	Wrong treatment	3.0	5.0	4.7	70.0

Schedule plan	Imaging delay at console	Imaging scheduled in ARIA (cannot be modified)	Treatment delay	2.0	2.0	2.0	8.0
	Planned/scheduled fractions do not match	Too many fractions scheduled	Inconsistent record	2.0	2.3	2.0	9.3
Approve for treatment	Error missed	Warning screen misinterpreted (new warning mistaken as normal)	Wrong treatment	3.3	3.7	4.3	53.0
Schedule treatment	Planned/scheduled fractions do not match	Treatment not scheduled	Treatment delay	3.3	2.0	1.3	8.9
		Wrong linac selected	Treatment delay	2.7	2.3	2.0	12.4
		Wrong appointment type scheduled	Treatment delay	2.7	2.3	2.7	16.6
OPTIONAL fraction #2: increase reference dose	Incorrect reference dose	Reference dose not increased	Treatment delay	2.7	2.3	2.3	14.5
		Reference dose changed to wrong value	Wrong treatment	2.7	2.3	2.3	14.5
Section 3: Therapist Pre-Treatment							
Daily QA performed	Mechanical error, photon output, or imaging/isocenter deviation missed	Incomplete DailyQA review	Wrong treatment	2.0	6.0	4.3	52.0
	Safety interlock malfunction missed	Incomplete DailyQA review	Patient injury/Unintended radiation exposure	1.7	6.0	3.7	36.7
Cover linac with plastic protection	Plastic protection ineffective	Clear bed covers not completely secured to couch, controls, or gantry head	COVID-19 contamination	2.3	4.7	4.3	47.2

		Hand pendants used outside of plastic cover	COVID-19 contamination	2.3	4.7	4.3	47.2
		Equipment covers improperly secured to imagers	COVID-19 contamination	2.3	4.7	4.3	47.2
	Plastic protection interferes with gantry motion	Plastic cover not properly secured to gantry head	Collision	2.3	3.0	2.7	18.7
Place enhanced droplet isolation sign	Sign ineffective	Sign not placed in highly visible location	COVID-19 exposure	2.3	6.7	3.3	51.9
		Sign removed too early	COVID-19 exposure	2.0	6.7	4.0	53.3
		Untrained personnel bypasses sign without proper PPE	COVID-19 exposure	2.0	7.0	3.7	51.3
Move rails out	Rails not fully out during treatment	Rails left in wrong position	Wrong treatment	3.0	3.7	4.3	47.7
		Rails moved in during patient setup	Wrong treatment	3.7	3.7	4.3	58.3
Load treatment preplan at console	Incorrect plan loaded at console	Wrong patient selected	Wrong treatment	1.7	4.3	2.0	14.4
		Wrong treatment course selected	Wrong treatment	1.7	4.3	2.0	14.4
Add imaging at console	Image repeated (poor quality)	Wrong imaging protocol selected	Unintended radiation exposure	2.0	2.7	2.3	12.4
		Wrong techniques selected	Unintended radiation exposure	2.0	2.7	2.3	12.4
Don PPE at treatment linac	PPE ineffective	PPE not donned appropriately	COVID-19 exposure	3.3	6.7	5.3	118.5
		PPE breach missed	COVID-19 exposure	3.7	7.0	5.3	136.9

	Not enough PPE at linac	Lack of coordination	Treatment delay	3.0	2.7	2.0	16.0
"Green light" given to transfer patient to linac	Green light not delivered at correct time	Green light given too soon	COVID-19 exposure	3.0	3.3	2.3	23.3
		Green light given late	Treatment delay	3.0	2.7	1.7	13.3
			Section 4: Patient Setup & Pre-Treatment				
Patient transported to linac	Personnel exposed to COVID-19 positive patient	Wrong pathway taken	COVID-19 exposure	2.7	6.7	4.3	77.0
		Pathway not cleared and secured	COVID-19 exposure	2.7	6.7	4.3	77.0
		GK holding room occupied	COVID-19 exposure	2.3	5.7	2.7	35.3
Patient setup on table	Patient injured during setup	Patient injured during transfer	Patient injury	1.7	6.3	2.0	21.1
		Ventilator compromised during setup	Patient injury	2.3	7.7	2.7	47.7
	Patient position incompatible with linac motion	Clearance check not performed for angled patient	Collision	3.3	7.0	4.3	101.1
Align patient to isocenter	Patient not aligned	Patient grossly misaligned or moved after alignment	Treatment delay	3.0	5.0	3.3	50.0
		Patient angle improperly accounted for	Treatment delay	3.3	5.0	4.0	66.7
Perform timeout	Team members not present during emergency	Timeout did not verify presence of all team members	Patient injury	3.7	5.7	2.7	55.4

	ICU staff delayed in attending to acute distress	ICU staff not notified during timeout that doors can be opened to pause treatment for emergency	Patient injury	2.7	6.7	2.7	47.4
Monitor ventilator	Ventilator monitor failure	Camera on ventilator improperly set	Patient injury	3.0	5.3	2.3	37.3
		Ventilator improperly secured	Patient injury	2.7	5.7	3.3	50.4
		Ventilator power supply compromised	Patient injury	2.7	5.0	2.7	35.6
Image isocenter	Image repeated (poor quality)	Jaws and MLC settings not verified for image	Unintended radiation exposure	2.0	2.7	2.7	14.2
		Wrong techniques selected	Unintended radiation exposure	2.3	2.7	2.7	16.6
		MV imager height not properly adjusted	Unintended radiation exposure	2.7	4.0	4.0	42.7
	Patient not aligned to isocenter	Major alignment change not made following imaging	Wrong treatment	3.0	4.7	3.7	51.3
	Isocenter not set appropriately	Isocenter location not recorded	Wrong treatment	2.7	5.3	4.3	61.6
		Isocenter not set to midline/carina	Wrong treatment	2.3	5.0	3.3	38.9
Obtain extended CBCT	CBCT incompatible with gantry motion	MV imager not retracted before CBCT	Collision	3.3	5.0	3.0	50.0
		Clearance for CBCT not verified	Collision	3.0	5.0	3.0	45.0
	Image repeated (poor quality)	Patient improperly aligned for image	Unintended radiation exposure	2.0	2.7	2.3	12.4
		Scans do not capture full SUP-INF lung volume	Unintended radiation exposure	2.7	3.0	2.3	18.7

	Incorrect AP distance across lung	AP separation recorded incorrectly	Wrong treatment	2.3	6.3	5.0	73.9
Recenter table	Patient not aligned to isocenter	Couch moved to unintended isocenter	Wrong treatment	1.7	6.3	3.0	31.7
		Couch not moved following CBCT	Wrong treatment	1.7	6.3	3.0	31.7
Create empty treatment fields at console	Wrong treatment field created	Wrong field type selected (not static)	Wrong treatment	2.0	3.0	2.7	16.0
		Wrong MLC selection ("use open MLC" selected)	Wrong treatment	3.3	3.0	2.0	20.0
		Wrong beam parameters selected (dose rate, energy, gantry rotation)	Wrong treatment	3.3	4.0	3.0	40.0
	Temporary MU mistaken for planned MU	Wrong # of MUs selected	Wrong treatment	2.0	6.7	2.7	35.6
Create new portal image	Image repeated (poor quality)	Wrong energy/technique selected	Unintended radiation exposure	2.3	2.7	3.7	22.8
		Jaw settings too small to capture lung with margin	Unintended radiation exposure	2.3	2.7	2.3	14.5
		Wrong MV imager position recorded for AP field	Unintended radiation exposure	2.3	2.7	3.0	18.7
	Reference image incorrect	Portal not selected as reference	Treatment delay	2.3	2.7	3.0	18.7
	PA portal not compatible with gantry position	Imager not retracted to 50 cm for PA field	Collision	2.3	5.3	3.3	41.5
Acquire portal images for treatment planning	Portal image not delivered	Mechanical/console failure	Treatment delay	1.7	4.0	1.7	11.1
Section 5: Dose Calculation & Treatment Planning							

Record AP/PA jaw positions	Not accurately recorded	Jaw position not recorded	Wrong treatment	2.3	6.3	2.0	29.6
		Jaw position with or without margin unclear	Wrong treatment	2.3	5.0	4.7	54.4
Assign trial and dose level	Incorrect trial and dose level assigned to plan	Wrong trial selected	Wrong treatment	2.7	7.0	5.0	93.3
		Wrong dose selected	Wrong treatment	2.7	7.0	5.0	93.3
Calculate MU for photon dose	Incorrect MU calculation	Wrong notebook selected	Wrong treatment	2.3	5.3	3.0	37.3
		Wrong beam settings (photon energy, dose, SSD/SAD)	Wrong treatment	2.3	6.3	3.7	54.2
		Wrong jaw settings used in calculation	Wrong treatment	2.7	6.3	3.7	61.9
		Wrong patient parameters (thickness, etc.)	Wrong treatment	2.7	5.7	3.7	55.4
		Isocenter not set to midline/carina	Wrong treatment	2.7	5.7	4.0	60.4
Assemble documents for treatment	Incorrect treatment information	Error missed in documentation	Wrong treatment	2.7	3.7	2.7	26.1
		Documents incomplete	Treatment delay	2.3	2.7	2.7	16.6
		Documents omitted	Inaccurate record	2.3	2.7	2.7	16.6
Section 6: Treatment Delivery							
Return couch to isocenter	Couch not moved to isocenter	Miscommunication/human error	Wrong treatment	2.0	6.7	2.3	31.1

Perform second check	Incorrect treatment parameters	Errors missed in second check	Wrong treatment	2.0	6.7	5.3	71.1
Add calculated MU to fields	Incorrect MU entered	MU not calculated correctly	Wrong treatment	2.3	6.7	5.3	83.0
		Entry error	Wrong treatment	2.3	6.7	3.3	51.9
Add MV portal image to treatment field	Positioning error missed	MV image not assigned to field	Wrong treatment	2.0	4.3	3.0	26.0
		Image misinterpreted (error not seen)	Wrong treatment	2.7	5.3	3.7	52.1
Apply MV image as reference before each field	Image not assigned as new reference	Human error	Inaccurate record	2.7	2.7	2.3	16.6
Treat	Beam delivery not completed	Beam interrupt triggered	Treatment delay	2.0	3.7	1.7	12.2
		Mechanical failure	Treatment delay	1.7	5.0	1.7	13.9
		Patient motion during delivery	Treatment delay	2.0	4.7	2.7	24.9
Take screenshot of final treatment delivery	Incomplete record of treatment delivery	Image not taken	Inconsistent record	2.3	2.7	3.3	20.7
Deliver "NoTx DoseRecord" field to write back data to ARIA	Data not written to record & verify system	"NoTx DoseRecord" field not delivered	Inaccurate record	3.3	2.7	3.0	26.7
		Data write-back error missed	Inaccurate record	2.0	2.7	3.0	16.0
Close patient at console	Data modified after treatment end	Patient chart not closed correctly	Inaccurate record	2.0	2.7	2.3	12.4
Section 7: Post-Treatment							

Remove patient from room for transport	Personnel exposed to COVID-19 positive patient	Wrong pathway taken	COVID-19 exposure	2.0	6.3	3.3	42.2
		Pathway not cleared and secured	COVID-19 exposure	2.0	6.3	3.3	42.2
	Patient injured during transfer	Lack of training/human error	Patient injury	2.0	7.0	1.7	23.3
		Ventilator compromised during transfer	Patient injury	1.7	7.7	2.0	25.6
Contact EVS dispatch for terminal cleaning	Terminal cleaning not scheduled	EVS not contacted/lack of coordination	COVID-19 contamination	2.3	7.3	4.7	79.9
Remove plastic cover from couch	Subsequent patient exposed to contaminated surface	Plastic protection not removed between patients	COVID-19 contamination	2.0	7.3	2.3	34.2
Wipe down or remove plastic covers on gantry/imaging	Plastic protection ineffective	Plastic protection removed too early	COVID-19 contamination	2.7	6.3	3.0	50.7
		Wipe down ineffective	COVID-19 contamination	1.7	8.0	7.0	93.3
Close room doors	Personnel exposed to high risk COVID-19 environment	Doors not closed following patient exit	COVID-19 exposure	2.0	7.0	2.7	37.3
Doff PPE	PPE ineffective	PPE doffed too early	COVID-19 exposure	3.3	7.3	3.7	89.6
		PPE not doffed appropriately	COVID-19 contamination	3.7	8.0	4.3	127.1
Determine time for EVS cleaning (>69 minutes after patient leaves)	Incorrect time determined	Time calculation forgotten	COVID-19 exposure	2.3	6.3	5.7	83.7
		Calculation error	COVID-19 exposure	2.3	6.3	5.7	83.7

Place contact isolation sign with time for EVS entry	Sign ineffective	Sign not placed in highly visible location	COVID-19 exposure	3.3	7.3	5.7	138.5
		Sign removed too early	COVID-19 exposure	2.0	7.3	6.0	88.0
After EVS: disinfect couch, controls, and hand pendants	Wipe down ineffective	Wipe down forgotten	COVID-19 contamination	3.3	8.0	7.0	186.7
		Wipe down performed incorrectly (lack of training)	COVID-19 contamination	3.3	8.0	7.7	204.4
Verify data has been written back to ARIA	Error in data transfer missed	Incomplete verification	Inaccurate record	2.0	2.0	2.3	9.3
Close out patient chart following standard protocol	Closeout performed incorrectly	Lack of training/human error	Inconsistent record	2.0	2.0	2.7	10.7
