# Supplementary material for: Multi‐phase failure modes and effects analysis for low dose bilateral whole lung irradiation of COVID‐19 positive patients requiring respiratory ventilation
Source: J Appl Clin Med Phys. 2024 Jan 9;25(4):e14261. doi: 10.1002/acm2.14261 (PMC11005974; doi:10.1002/acm2.14261)
Supplement: Supplementary file 2 — Supporting Information [file ACM2-25-e14261-s001.pdf]

Supplemental Table 2: Median of individual scores determined for the full Phase II survey (†), group scores determined during the re-scoring session (‡), and observed differences in scores between the two methods. Descriptions for process steps that were rephrased during the group scoring session are marked to identify which version was provided in each method (†,‡).

Process Step	***Potential Failure Mode***	***Potential Cause of Failure***	***End Effect***	*O*	*S*	*D*	*RPN*	*O*	*S*	*D*	*RPN*	*O*	*S*	*D*	*RPN*
				Median scores†				Group scores (‡)				Difference (‡-†)			
Obtain patient consent for trial	Contraindication for treatment missed	Incomplete history obtained	Patient injury	2	6	5	**60**	2	6	5	**60**	0	0	0	**0**
Enter patient in REDCap for research tracking	Wrong patient entered into REDCap	Human error	Wrong patient	2	4	3	**24**	5	1	3	**15**	3	-3	0	**-9**
Coordinate treatment time and notify teams in RadOnc and Respiratory Care	Department not notified	Text not received (no signal, device down)	Treatment delay	3	3	4	**36**	3	3	7	**63**	0	0	3	**27**
Create course and plan from template	Course/plan information inaccurate	Wrong energy manually selected	Wrong treatment	3	6	3	**54**	3	4	3	**36**	0	-2	0	**-18**
Prescribe dose	Preplan dose not 80 cGy	Dose incorrectly entered	Wrong treatment	3	7	3	**63**	4	7	3	**84**	1	0	0	**21**
Submit for plan approval	Error missed	Incomplete preplan review	Wrong treatment	3	6	5	**90**	3	6	5	**90**	0	0	0	**0**
Place enhanced droplet isolation sign	Sign ineffective	Sign removed too early	COVID-19 airborne exposure	3	7	5	**105**	3	4	5	**60**	0	-3	0	**-45**
Move rails out	Rails not fully out during treatment	Rails moved in during patient setup	Wrong treatment	3	4	3	**36**	3	3	1	**9**	0	-1	-2	**-27**

Don PPE at treatment linac	PPE ineffective	PPE not donned appropriately or PPE breach missed	COVID-19 airborne exposure	3	7	5	**105**	4	4	6	**96**	1	-3	1	**-9**
Patient transported to linac	Personnel exposed to COVID-19 positive patient	Pathway not clearer and secured, or wrong pathway taken	COVID-19 airborne exposure	4	6	4	**96**	4	7	4	**112**	0	1	0	**16**
Patient setup on table	Patient injured during setup	Ventilator compromised during setup	Patient injury	2	8	2	**32**	2	8	2	**32**	0	0	0	**0**
	Patient position incompatible with linac motion	Clearance check not performed for angled patient	Collision	2	8	3	**48**	2	8	3	**48**	0	0	0	**0**
Align patient to isocenter	Patient not aligned	Patient angle improperly accounted for	Treatment delay	3	3	3	**27**	3	5	3	**45**	0	2	0	**18**
Perform timeout	Team members not present during emergency	Timeout did not verify presence of all team members or was not performed	Patient injury	3	7	2	**42**	3	7	2	**42**	0	0	0	**0**
Image isocenter	Isocenter not set appropriately	Isocenter location not recorded	Wrong treatment	3	7	3	**63**	3	6	3	**54**	0	-1	0	**-9**
Obtain extended CBCT	Incorrect AP distance across lung	AP distance recorded incorrectly	Wrong treatment	3	6	4	**72**	3	6	4	**72**	0	0	0	**0**
Record AP/PA jaw positions	Not accurately recorded	Jaw position with or without margin unclear	Wrong treatment	3	6	4	**72**	3	5	4	**60**	0	-1	0	**-12**
†*Assign trial and dose level* // ‡*Assign dose to plan*	†Incorrect trial and dose level assigned to plan // ‡Preplan dose not 80 cGy	†Wrong dose or trial selected // ‡Dose incorrectly entered	†Wrong treatment // ‡Wrong treatment	2	7	3	**42**	2	7	3	**42**	0	0	0	**0**
Calculate MU for photon dose	Incorrect MU calculation	Wrong jaw settings or patient parameters	Wrong treatment	3	7	4	**84**	3	4	4	**48**	0	-3	0	**-36**

		(thickness, etc.) used in calculation													
		†Isocenter not set to midline/carina // ‡Wrong treatment depth (not derived from isocenter)	Wrong treatment	3	6	3	**54**	**3**	3	3	**27**	0	-3	0	**-27**
Perform second check	Incorrect treatment parameters	Errors missed in second check	Wrong treatment	2	7	5	**70**	2	5	6	**60**	0	-2	1	**-10**
Add calculated MU to fields	Incorrect MU entered	MU calculated or entered incorrectly	Wrong treatment	3	7	4	**84**	3	6	4	**72**	0	-1	0	**-12**
Add MV portal image to treatment field	Positioning error missed	Image misinterpreted (error not seen)	Wrong treatment	3	7	4	**84**	2	4	2	**16**	-1	-3	-2	**-68**
Remove patient from room for transport	Patient injured during transfer	Ventilator compromised during transfer	Patient injury	2	8	2	**32**	2	8	2	**32**	0	0	0	**0**
Contact EVS dispatch for terminal cleaning	Terminal cleaning not scheduled	EVS not contacted/lack of coordination	COVID-19 surface contamination	3	6	6	**108**	2	6	6	**72**	-1	0	0	**-36**
Wipe down or remove plastic covers on gantry/imaging	Plastic protection ineffective	Wipe down ineffective	COVID-19 surface contamination	3	6	8	**144**	2	6	7	**84**	-1	0	-1	**-60**
Doff PPE	PPE ineffective	PPE doffed too early or not doffed appropriately	COVID-19 airborne exposure	3	7	5	**105**	4	7	5	**140**	1	0	0	**35**
Determine time for EVS cleaning (>69 minutes after patient leaves)	Incorrect time determined	Time calculation forgotten or performed incorrectly	COVID-19 airborne exposure	2	5	5	**50**	2	5	5	**50**	0	0	0	**0**

Place contact isolation sign with time for EVS entry	Sign ineffective	Sign not placed in highly visible location or removed too early	COVID-19 airborne exposure	3	6	3	**54**	2	5	3	**30**	-1	-1	0	**-24**
After EVS: disinfect couch, controls, and hand pendants	Wipe down ineffective	Wipe down forgotten or performed incorrectly	COVID-19 surface contamination	3	6	8	**144**	3	4	8	**96**	0	-2	0	**-48**
